# Seroprevalence of anti-SARS-CoV-2 IgG antibodies, risk factors for infection and associated symptoms in Geneva, Switzerland: a population-based study

**DOI:** 10.1177/14034948211048050

**Published:** 2021-10-19

**Authors:** Aude Richard, Ania Wisniak, Javier Perez-Saez, Henri Garrison-Desany, Dusan Petrovic, Giovanni Piumatti, Hélène Baysson, Attilio Picazio, Francesco Pennacchio, David De Ridder, François Chappuis, Nicolas Vuilleumier, Nicola Low, Samia Hurst, Isabella Eckerle, Antoine Flahault, Laurent Kaiser, Andrew S. Azman, Idris Guessous, Silvia Stringhini

**Affiliations:** 1 Division of Primary Care, Geneva University Hospitals, Switzerland; 2 Institute of Global Health, University of Geneva, Switzerland; 3 Department of Epidemiology, Johns Hopkins Bloomberg School of Public Health, USA; 4 University Centre for General Medicine and Public Health (UNISANTE), University of Lausanne, Switzerland; 5 Faculty of BioMedicine, Università della Svizzera Italiana, Switzerland; 6 Department of Health and Community Medicine, University of Geneva, Switzerland; 7 Division of Tropical and Humanitarian Medicine, Geneva University Hospitals, Switzerland; 8 Division of Laboratory Medicine, Geneva University Hospitals, Switzerland; 9 Department of Medicine, University of Geneva, Switzerland; 10 Institute of Social and Preventive Medicine, University of Bern, Switzerland; 11 Institute for Ethics, History, and the Humanities, University of Geneva, Switzerland; 12 Geneva Center for Emerging Viral Diseases and Laboratory of Virology, Geneva University Hospitals, Switzerland; 13 Department of Microbiology and Molecular Medicine, University of Geneva, Switzerland; 14 Division of Infectious Diseases, Geneva University Hospitals, Switzerland

**Keywords:** SARS-CoV-2, COVID-19, population-based survey, seroprevalence, socioeconomic risk factors

## Abstract

***Aims:*** To assess SARS-CoV-2 seroprevalence over the first epidemic wave in the canton of Geneva, Switzerland, as well as risk factors for infection and symptoms associated with IgG seropositivity. ***Methods:*** Between April and June 2020, former participants of a representative survey of the 20–74-year-old population of canton Geneva were invited to participate in the study, along with household members aged over 5 years. Blood samples were tested for anti-SARS-CoV-2 immunoglobulin G. Questionnaires were self-administered. We estimated seroprevalence with a Bayesian model accounting for test performance and sampling design. ***Results:*** We included 8344 participants, with an overall adjusted seroprevalence of 7.8% (95% credible interval 6.8–8.9). Seroprevalence was highest among 18–49 year-olds (9.5%), and lowest in 5–9-year-old children (4.3%) and individuals >65 years (4.7-5.4%). Odds of seropositivity were significantly reduced for female retirees and unemployed men compared to employed individuals, and smokers compared to non-smokers. We found no significant association between occupation, level of education, neighborhood income and the risk of being seropositive. The symptom most strongly associated with seropositivity was anosmia/dysgeusia. ***Conclusions:* Anti-SARS-CoV-2 population seroprevalence remained low after the first wave in Geneva. Socioeconomic factors were not associated with seropositivity in this sample. The elderly, young children and smokers were less frequently seropositive, although it is not clear how biology and behaviours shape these differences.**

## Introduction

Seroprevalence studies around the world have established that only a small proportion of the population had been infected by SARS-CoV-2 towards the end of the first wave in spring 2020, with most seroprevalence estimates ranging from less than 0.1 to more than 20%, depending on the setting and targeted populations [1–3]. Preliminary results from the first 5 weeks of our 12-week population-based serosurvey conducted in the canton of Geneva, Switzerland, in April and May, showed a weekly seroprevalence between 4.8% and 10.9% [4]. Regions with similar clinical incidence rates in France, Italy and Spain showed similar results [5–7], while the least affected regions in Spain and France showed seroprevalences of less than 3% [5–8].

Socioeconomic risk factors for COVID-19 such as low income, social deprivation and overcrowded living conditions have been identified in several studies based on molecular testing [9–13]. However, some of those studies included only hospitalised patients or individuals with severe COVID-19. In addition, data on confirmed COVID-19 cases are influenced by testing policies and comprise only a minority of the whole infected population. Serosurveys have the potential to assess the real extent of an outbreak in a given geographical region and provide information on risk factors, transmission and infection fatality rates without relying on clinical surveillance and virological confirmation. Nevertheless, only a few population-based serostudies have assessed biological and socioeconomic risk factors for seropositivity. These have generally highlighted significant relationships between Black, Asian and Hispanic ethnicity, adult age (varying across studies), living in a deprived or dense area and large household size, and the risk of being seropositive [5–8, 14, 15]. While working in the healthcare field or essential services has been shown to be associated with a higher risk of seropositivity [6, 7, 14, 16], other individual factors such as education level and occupational category are rarely described. Serosurveys can also provide a means to quantify the full spectrum of symptoms associated with SARS-CoV-2, including mild and asymptomatic infections.

In Switzerland, the first cases of COVID-19 were recorded at the end of February 2020, followed by a first epidemic wave that took place during March and April 2020 [17]. Strict non-pharmaceutical measures started on 16 March and were progressively relaxed from 27 April 2020 when a total of 29,313 cases and 427 deaths had been reported in the country [18]. In this study, we describe the age-specific seroprevalence of anti-SARS-CoV-2 antibodies between April and June 2020 in Geneva, Switzerland, and examine risk factors for and symptoms associated with seropositivity.

## Methods

### Study design and participants

SEROCoV-POP is a population-based repeated cross-sectional observational seroprevalence study. We recruited participants between 6 April and 30 June 2020. The recruitment strategy and serological data collection have previously been described in detail [4]. Briefly, participants were invited progressively by email and postal mail from the study population of a yearly health survey called Bus Santé [19], which is representative of the 20–74-year-old population of canton Geneva. Participants were invited to bring members of their household aged 5 years and older. Each person participated only once (one round). Of note, during the study period, the country was initially under lockdown; then, measures were progressively relaxed in three consecutive periods [20]. Written informed consent was obtained from all individual participants included in the study and from legal guardians for children under 18 years of age. The study protocol was approved by the Cantonal Research Ethics Commission of Geneva, Switzerland (CER16-363) and is available online at https://static1.squarespace.com/static/5e7dd8f02d3bc353fbb05121/t/5e887016c7fa18312c3e00fa/1585999900784/Protocole_SEROCOV-POP30.03.pdf.

### Laboratory analysis

Anti-SARS-CoV-2 immunoglobulin G (IgG) serological status was assessed using a commercially available enzyme-linked immunosorbent assay (ELISA) (Euroimmun; Lübeck, Germany #EI 2606-9601 G) targeting the S1 domain of the spike protein. We used the manufacturer’s recommended cut-off for positivity (⩾1.1), with a reported sensitivity of 93% and a specificity of 100% estimated in a diagnostic validation study conducted in the same lab as these analyses [21]. We tested all serum with an ELISA IgG ratio of 0.5 or greater with an immunofluorescence assay (IFA) and classified individuals with results from this test (when performed) in sensitivity analyses.

### Survey questions and coding

Participants could choose to fill in the study questionnaire online at home or complete it at the study site on a tablet computer or on paper. Questionnaires included items about sociodemographic characteristics, symptoms, exposure to SARS-CoV-2-infected individuals and preventive behaviours in relation to the epidemic. Sociodemographic variables included age, sex, employment status, professional occupation, professional changes due to the pandemic and educational level. Educational level was based on the national Swiss system, with ‘mandatory education’ applying to all children aged 4–14 years, ‘secondary education’ corresponding to high school (15–18 years) and ‘university’ corresponding to undergraduate and postgraduate studies.

Participants were also asked to self-identify their occupational sector and profession. We mapped this information using the European socioeconomic classifications (ESECs). From the 10 levels of classification, in order to maintain power, this was reduced to eight categories: (a) professional/manager (ESEC 1–2); (b) higher grade white-collar workers (ESEC 2); (c) independent workers (ESEC 4–5); (d) lower grade white-collar workers(ESEC 7); (e) blue-collar, skilled, semi-skilled and non-skilled workers (ESEC 6, 8, 9); (f) other full-time workers to whom the ESEC categories were not applicable; as well as from ESEC class 10; (g) students or retirees; and (h) unemployed.

Participants were asked to self-report all symptoms unrelated to a known chronic condition experienced since January 2020, and to specify if these appeared as part of one or several distinct episodes. We defined participants as asymptomatic if they did not report any symptoms. Data were also collected on chronic health conditions, smoking status and height and weight for body mass index (BMI) calculation. The full study questionnaire is provided in the Supplemental material.

### Other data sources

Data on individual-level income was not collected in the questionnaire. However, we used data from the canton of Geneva to determine the median income for a single individual residing in participants’ neighbourhoods. We grouped gross income into three categories defined as less than 37,000 CHF/year (70% of the median income), 37,000–68,000 CHF/year and more than 68,000 CHF/year (~130% of the median income) [22].

### Statistical analysis

We estimated the overall 12-week seroprevalence using a Bayesian logistic regression model accounting for test performance, sex, age as well as within household infection clustering, as previously described [4]. In our primary analyses, we used IgG ELISA results only to classify seropositivity, and performed post-stratification to obtain population-wide seroprevalence as well as by age and sex classes. As a sensitivity analysis, we used the IFA results for all participants tested with this assay. Models were implemented in the Stan probabilistic programming language. Details are provided in the Supplemental material. Weekly crude proportions of seropositivity with binomial confidence intervals (CIs) were also calculated.

For socioeconomic and lifestyle risk factors, we limited our analysis to participants 18 years of age or older, resulting in a final analytical sample of 7442 participants. Estimated marginal proportions were adjusted for age and sex, with significance testing using the Tukey method [23] for comparing estimates between seronegative and seropositive groups. To test for association between sociodemographic, lifestyle and health-related risk factors and serological status, we used mixed effect logistic models including a household-level random effect, except for the area-level income analysis. To understand the sensitivity of our results to missing data, we conducted multilevel multiple imputation by chained equations using 10 datasets with 30 iterations across the dataset and re-ran our primary analyses.

Chi-square tests were used to compare the frequencies of individual symptoms between IgG seropositive and seronegative individuals. Using logistic regression models, we estimated odds ratios (ORs) and 95% CIs for IgG seropositivity according to the presence of each symptom in the overall study sample and stratified by age groups. As a sensitivity analysis, we re-ran the logistic regression on individuals presenting only one symptomatic episode. To take into account the co-occurrence of symptoms, we fit multivariable logistic regression models to estimate sex-adjusted and mutually adjusted ORs. The absence of a symptom was defined as the reference value for each symptom, while for the asymptomatic variable, not being asymptomatic was considered the reference value. Analyses were done using R statistical software version 4.0.3 [24].

## Results

Between 6 April and 30 June 2020, we enrolled 8344 participants between the ages of 5 and 94 years (902 children <18 years), with a mean age of 46.9 years. The study sample included 4379 former Bus Santé participants and 3965 household members, with a 42% participation rate of all people invited by email (Supplemental Figure 1).

Compared with the population of the canton of Geneva, in SEROCoV-POP, the 50–74 years age group was overrepresented (46.6% vs. 27.4%), while the 5–9, 20–49 and 75 years and over age groups were slightly underrepresented. SEROCoV-POP participants also had generally higher levels of education than the Geneva population, with more participants having received a tertiary education (57.7% vs. 39.3%), and fewer having attended mandatory school only (6.8% vs. 28.1%) (Supplemental Table 1).

### Seroprevalence and relative risk of seropositivity by age and sex

SARS-CoV-2 seroprevalence over the study period was 7.8% (95% credible interval (CrI) 6.8–8.9, Table I). The seroprevalence first increased from 7.1% (95% CrI 5.5–8.7) to 9.0% (95% CrI 7.5–10.5) between the first and second month, before decreasing again to 7.1% (95% CrI 5.7–8.5) in the third month (Table I).

**Table I.** Seroprevalence estimates and relative risk of SARS-CoV-2 seropositivity (*N*=8344).

	SARS-CoV-2 IgG test result		Seroprevalence (95% credible interval)^a^	Relative risk (95% credible interval)	*P* value
	Positive	Negative			
Age group, years					
<10 (*n*=274)	9 (3.3%)	265 (96.7%)	4.3 (2.2–7.0)	0.44 (0.22–0.72)	<0.001
10–17 (*n*=628)	50 (8.0%)	578 (92.0%)	7.9 (5.9–10.3)	0.83 (0.62–1.09)	0.1752
18–49 (*n*=3108)	278 (9.0%)	2830 (91.1%)	9.5 (8.1–10.9)	1 (ref)	–
50–64 (*n*=2694)	180 (6.7%)	2514 (93.3%)	7.5 (6.2–8.8)	0.79 (0.65–0.94)	0.0076
65–74 (*n*=1196)	56 (4.7%)	1140 (95.3%)	5.4 (4.0–7.0)	0.57 (0.42–0.76)	<0.001
>75 (*n*=444)	17 (3.8%)	427 (96.2%)	4.7 (2.8–7.0)	0.50 (0.29–0.75)	0.0012
Sex					
Female (*n*=4465)	285 (6.4%)	4180 (93.6%)	7.0 (6.0–8.1)	1 (ref)	–
Male (*n*=3879)	305 (7.9%)	3574 (92.1%)	8.7 (7.4–10.0)	1.2 (1.1–1.4)	0.007
Month					
April (*n*=1939)	130 (6.7%)	1809 (93.3%)	7.1 (5.5–8.7)		0.052
May (*n*=2997)	244 (8.1%)	2753 (91.9%)	9.0 (7.5–10.5)		–
June (*n*=3408)	216 (6.3%)	3192 (93.7%)	7.1 (5.7–8.5)		0.028
Overall (*n*=8344)	590 (7.1%)	7754 (92.9%)	7.8 (6.8–8.9)	–	–

a Calculated using a Bayesian logistic regression model accounting for test performance, sex and household clustering.

Men were 22% (95% CrI 6–40%) more likely to be seropositive than women. Seroprevalence was highest in the 18–49 years age group (9.5%, 95% CrI 8.1–10.9) and lowest in children under 10 years (4.3%, 95% CrI 2.2–7.0) and the elderly aged 75 years and older (4.7%, 95% CrI 2.8–7.0).

### Socioeconomic, lifestyle and health-related risk factors

After adjusting for age and sex, there was a significant difference in the distribution of seropositive participants according to employment status (Table II), with lower odds of being seropositive in female retirees (OR 0.46, 95% CI 0.23–0.93) and unemployed men (OR 0.35, 95% CI 0.13–1.0) compared to employed individuals of the same sex. However, we did not observe any significant association between occupational category, educational level, area-based income and seropositivity.

**Table II.** Sociodemographic, behavioural and health characteristics of the adult SEROCOV-POP study participants (*N*=7329) by SARS-CoV-2 serology test result and association with serological status.

	Overall	SARS-CoV-2 IgG test result		*P* value	OR (95% CI)^a^		
		Negative	Positive		Overall sample^1^	Women^2^	Men^2^
Employment status				**<0.001**			
Employed	3592 (49.7%)	3305 (92.0%)	286 (8.0%)		[Reference]	[Reference]	[Reference]
Unemployed	549 (7.6%)	516 (94.0%)	33 (6.0%)		0.62 (0.36–1.04)	0.86 (0.46–1.63)	**0.35 (0.13–1.00)**
Student	666 (9.2%)	596 (89.5%)	70 (10.5%)		0.81 (0.48–1.35)	1.2 (0.61–2.50)	0.56 (0.26–1.22)
Retired	1635 (22.6%)	1565 (95.7%)	70 (4.3%)		**0.61 (0.37–0.99)**	**0.46 (0.23–0.93)**	0.95 (0.50–1.81)
Freelance/other	781 (10.8%)	714 (91.4%)	67 (8.6%)		1.2 (0.80–1.81)	1.7 (0.90–3.13)	1.0 (0.56–1.79)
Occupational category				0.112			
Professionals/managers	743 (10.3%)	680 (91.5%)	63 (8.5%)		[Reference]	[Reference]	[Reference]
Higher-grade white collar worker	1136 (15.7%)	1048 (92.3%)	88 (7.7%)		0.80 (0.49–1.3)	1.6 (0.74–3.6)	**0.50 (0.25–1.00)**
Independents	161 (2.2%)	150 (93.2%)	11 (6.8%)		0.69 (0.27–1.8)	1.5 (0.34–6.8)	0.52 (0.15–1.8)
Lower-grade white collar worker	921 (12.7%)	846 (91.9%)	75 (8.1%)		1.0 (0.60–1.7)	1.7 (0.77–3.8)	0.79 (0.37–1.7)
Blue collar workers	368 (5.1%)	342 (92.9%)	26 (7.1%)		0.61 (0.31–1.2)	0.85 (0.24–3.0)	0.59 (0.26–1.4)
Other or N/A	1064 (14.7%)	974 (91.5%)	90 (8.5%)		1.0 (0.62–1.7)	2.0 (0.88–4.4)	0.61 (0.31–1.2)
Change in work				0.88			
None	1586 (32.2%)	1464 (92.3%)	122 (7.7%)		[Reference]	[Reference]	[Reference]
Any	3343 (67.8%)	3079 (92.1%)	264 (7.9%)		0.95 (0.68–1.3)	1.0 (0.75–1.4)	0.94 (0.58–1.5)
Specific change in work^3^				**<0.001**	[Reference: no changes]	[Reference: no changes]	[Reference: no changes]
Stopped activities	547 (11.1%)	506 (92.5%)	41 (7.5%)		0.87 (0.51–1.5)	0.97 (0.58–1.6)	0.90 (0.41–2.0)
Telework	2122 (43.0%)	1961 (92.4%)	161 (7.6%)		0.87 (0.61–1.3)	0.93 (0.65–1.3)	0.93 (0.56–1.6)
Took sick leave	48 (1.0%)	32 (66.7%)	16 (33.3%)		**18.9 (5.8–61.6)**	**7.3 (3.3–15.9)**	**10.8 (1.4–83.4)**
Unemployed	165 (3.3%)	155 (93.9%)	10 (6.1%)		0.58 (0.22–1.5)	0.95 (0.39–2.3)	0.43 (0.11–1.7)
Other	461 (9.4%)	425 (92.2%)	36 (7.8%)		1.06 (0.60–1.9)	1.03 (0.59–1.8)	0.99 (0.43–2.3)
Educational level				0.39			
Mandatory education only	466 (6.4%)	439 (694.2%)	27 (5.8%)		0.98 (0.43–2.2)	1.6 (0.40–6.6)	1.1 (0.37–3.1)
Apprenticeship	1106 (15.1%)	1044 (94.4%)	62 (5.6%)		1.2 (0.58–2.5)	2.2 (0.60–7.8)	1.0 (0.41–2.4)
Secondary education	1341 (18.3%)	1238 (92.3%)	103 (7.7%)		1.3 (0.67–2.7)	2.4 (0.71–8.3)	1.3 (0.53–3.1)
University	3576 (48.8%)	3285 (91.9%)	291 (8.1%)		1.7 (0.88–3.2)	3.0 (0.92–9.9)	1.4 (0.65–3.2)
Doctoral education	399 (5.4%)	378 (94.7%)	21 (5.3%)		[Reference]	[Reference]	[Reference]
Other	344 (4.7%)	321 (93.3%)	23 (6.7%)		1.3 (0.54–3.1)	2.9 (0.71–1.9)	0.81 (0.25–2.7)
Missing	97 (1.3%)	93 (95.9%)	4 (4.1%)		–	–	–
Neighbourhood income – single (CHF)				**0.02**			
Lowest (<37K CHF/year)	386 (5.2%)	357 (92.5%)	29 (7.5%)		[Reference]	[Reference]	[Reference]
Middle (37K to 68K CHF/year)	6705 (90.1%)	6236 (93.0%)	469 (7.0%)		0.91 (0.63–1.4)	1.3 (0.69–2.6)	0.73 (0.46–1.2)
Highest (>68K CHF/year)	244 (3.3%)	225 (92.2%)	19 (7.8%)		1.0 (0.55–1.9)	1.4 (0.55–3.7)	0.81 (0.35–1.8)
Missing	107 (1.4%)	93 (86.9%)	14 (13.1%)		–	–	–
Smoking				**<0.001**			
Never	4852 (66.2%)	4456 (91.8%)	396 (8.2%)		Reference	Reference	Reference
Former	1254 (17.1%)	1171 (93.4%)	83 (6.6%)		0.82 (0.58–1.2)	0.72 (0.47–1.1)	1.0 (0.63–1.7)
Current	1112 (15.2%)	1065 (95.8%)	47 (4.2%)		**0.36 (0.23–0.55)**	**0.40 (0.24–0.67)**	**0.38 (0.21–0.68)**
Missing	111 (1.5%)	106 (95.5%)	5 (4.5%)		–	–	–
BMI category^4^				0.84			
Underweight	385 (5.3%)	359 (92.5%)	26 (7.5%)		0.57 (0.10–1.04)	0.74 (0.37–1.5)	0.16 (0.02–1.2)
Normal weight	4170 (56.9%)	3857 (93.2%)	313 (6.8%)		Reference	Reference	Reference
Overweight	2033 (27.7%)	1893 (93.1%)	140 (6.9%)		1.0 (0.73–1.4)	0.83 (0.50–1.4)	1.0 (0.69–1.6)
Obese	655 (8.9%)	606 (92.5%)	49 (7.5%)		1.4 (0.85–2.2)	1.9 (0.95–3.7)	0.76 (0.39–1.5)
Missing	86 (1.2%)	83 (96.5%)	3 (3.5%)		–	–	–
Chronic health conditions				0.02			
None	5152 (69.9%)	4722 (92.1%)	403 (7.9%)		Reference	Reference	Reference
One chronic condition	1505 (20.5%)	1416 (94.1%)	89 (5.9%)		0.81 (0.58–1.2)	0.76 (0.46–1.3)	0.89 (0.55–1.5)
Two or more chronic conditions	603 (8.2%)	567 (94.0%)	36 (6.0%)		0.96 (0.57–1.6)	1.7 (0.81–3.7)	0.63 (0.30–1.3)
Missing	96 (1.3%)	93 (96.9%)	3 (3.1%)		–	–	–
Risk-contact exposure^5^				**<0.001**			
Exposure	546 (6.5%)	419 (76.7%)	127 (23.3%)		**4.8 (3.8–6.0)**	**5.3 (3.9–7.1)**	**4.3 (3.1–6.0)**
No exposure	7087 (84.9%)	6664 (94.0%)	423 (6.0%)		Reference	Reference	Reference

OR: odds ratio; CI: confidence interval; N/A: not applicable; CHF: Swiss Francs.

Estimated marginal proportions were adjusted for age and sex, with significance testing using the Tukey method for comparing estimates between seronegative and seropositive groups.

a Calculated using a mixed effect logistic model including a household-level random effect, except for the area-level income analysis. Significant values at the *P*<0.05 level are in bold.

1 Adjusted for age and sex.

2 Adjusted for age.

3 The reference category for the logistic model was self-reported ‘no changes in work’.

4 Underweight: body mass index <18.5 kg/m^2^; normal weight ⩾18.5 to <25 kg/m^2^; overweight ⩾25 to <30 kg/m^2^; obese ⩾30 kg/m^2^.

5 Risk-contact exposure was assessed in the overall study sample, including children.

Overall, 67.8% of the participants reported some change in work linked to the COVID-19 epidemic, such as working remotely (43.0%), taking a sick leave (1.0%) or becoming unemployed (3.3%). Sixteen out of 48 individuals who took sick leave were seropositive, suggesting that COVID-19 had been responsible for up to one-third of all sick leaves during the first wave.

There seemed to be an association of education with seropositivity in women, with a tendency towards increasing odds of seropositivity from mandatory schooling only (OR 1.6, 95% CI 0.40–6.6) to university level (OR 3.0, 95% CI 0.92–9.9), compared to women with a doctoral education.

We observed a gradient in seroprevalence between never smokers (8.2%), former smokers (6.6%) and current smokers (4.2%). However, only current smokers were significantly less likely to be seropositive compared to never smokers (OR 0.36, 95% CI 0.23–0.55). There was no significant association between chronic health conditions and seropositivity. Underweight individuals seemed to have decreased odds of seropositivity compared to individuals with a normal BMI (OR 0.57, 95% CI 0.10–1.04), although this trend was mostly apparent in men (OR 0.16, 95% CI 0.02–1.2 vs. 0.74 in women, 95% CI 0.37–1.5). On the other hand, obese women tended to have higher odds of seropositivity compared to women with a normal BMI (OR 1.9, 95% CI 0.95–3.7), while there seemed to be no difference in male participants (OR 0.76, 95% CI 0.39–1.5).

A history of close contact with a SARS-CoV-2-infected person increased the odds of seropositivity nearly fivefold (OR 4.8, 95% CI 3.8–6.0).

### Symptoms reported over one or more episodes

The symptoms most frequently reported by seropositive participants were fatigue (57% of seropositive participants), headache (52%), sneezing and/or rhinorrhea (48%), fever (46%), cough (46%), anosmia and/or dysgeusia (44%), and myalgia and/or arthralgia (43%) (Table III). Seronegative individuals most frequently reported sneezing/rhinorrhea (32%), while only 3% reported loss of taste or smell. Thirteen per cent of seropositive participants reported no symptoms, versus 43% of seronegative participants. Not reporting any symptoms during the study period reduced by five the odds of being seropositive.

**Table III.** Frequency of symptoms reported in all age groups.

Symptom	SARS-CoV-2 IgG test result		*P* value
	Positive (*N*=590)^a^	Negative (*N*=7754)^b^	
Fatigue	332 (57%)	1787 (23%)	<0.001
Headache	308 (52%)	1936 (25%)	<0.001
Sneezing/rhinorrhea	284 (48%)	2468 (32%)	<0.001
Fever	272 (46%)	1136 (15%)	<0.001
Cough	270 (46%)	1790 (23%)	<0.001
Anosmia/dysgeusia	257 (44%)	233 (3.0%)	<0.001
Myalgia/arthralgia	252 (43%)	1160 (15%)	<0.001
Sore throat	200 (34%)	1786 (23%)	<0.001
Dyspnea	131 (22%)	577 (7.5%)	<0.001
Loss of appetite	126 (21%)	323 (4.2%)	<0.001
Diarrhoea	121 (21%)	749 (9.8%)	<0.001
Abdominal pain	65 (11%)	527 (6.9%)	<0.001
Nausea/vomiting	55 (9.4%)	374 (4.9%)	<0.001
Other symptoms	18 (3.1%)	181 (2.4%)	0.4
Asymptomatic	77 (13%)	3272 (43%)	<0.001

a Three missing values not included.

b 107 missing values not included. These participants had missing values for all symptoms, as they did not fill in the symptoms questionnaire.

**Figure 1.**
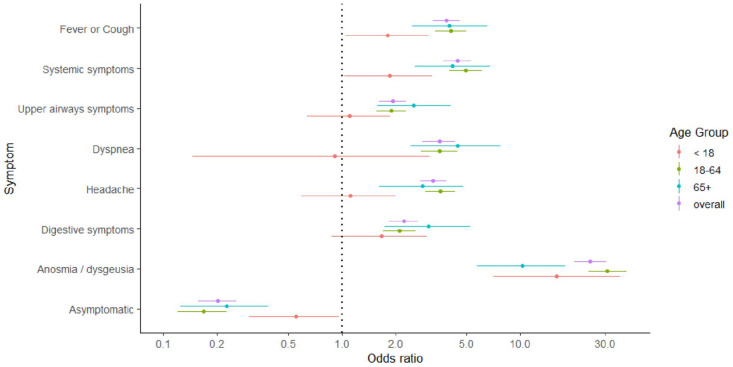
Age-stratified univariate odds ratios of seropositivity according to symptoms. Error bars correspond to 95% confidence intervals of odds ratios. Systemic symptoms: presence of either fatigue, myalgia, arthralgia or loss of appetite. Upper airways symptoms: presence of either sneezing/rhinorrhea, sore throat or both. Digestive symptoms: one or more of abdominal pain, nausea/vomiting and diarrhoea.

We observed age variations in the symptomatology associated with being seropositive. In children, anosmia and/or dysgeusia (OR 16.0, 95% CI 7.0–36.2), cough and/or fever (OR 1.8, 95% CI 1.1–3.1) and systemic symptoms (OR 1.9, 95% CI 1.0–3.2) were the groups of symptoms most strongly associated with seropositivity (Figure 1). On the contrary, among participants 65 years and older, dyspnea (OR 4.5, 95% CI 2.4–7.8), digestive symptoms (OR 3.1, 95% CI 1.7–5.2) and upper airways symptoms (OR 2.5, 95% CI 1.6–4.9) were more strongly associated with seropositivity than in the other age categories, although CIs overlap. In all age categories, those not reporting symptoms were less likely to be seropositive, although we observed a stronger association in the 18–64 years (OR 0.17, 95% CI 0.12–0.23) and over 65 years (OR 0.23, 95% CI 0.12–0.39) age groups than in children (OR 0.55, 95% CI 0.30–0.97).

In the multivariate analysis with co-adjustment of symptoms we observed that nausea and/or vomiting, abdominal pain and a sore throat were negatively associated with being seropositive (Supplemental Table 11).

Results for seroprevalence, risk factor and symptom analyses were mostly consistent when using the IFA confirmatory test (Supplemental Tables 3, 10, 13 and 14). The symptom analysis was also generally similar when considering only participants with only one symptomatic episode (Supplemental Tables 15 and 16).

## Discussion

This study conducted between April and June 2020 confirms preliminary results showing that fewer than 10% of the population of the canton of Geneva had developed anti-SARS-CoV-2 antibodies over the course of the first epidemic wave [4]. Consistent with our previously published interim analysis, individuals aged under 10 years and over 65 years, as well as women, had the lowest risk of being seropositive. We did not identify clear associations between socioeconomic variables and serological status. Smoking appeared to reduce the odds of being seropositive, while no significant associations were found with other lifestyle and health-related risk factors. The symptom most strongly associated with seropositivity was anosmia/dysgeusia, followed by systemic symptoms such as loss of appetite, fever, fatigue and myalgia and/or arthralgia.

The average seroprevalence of 7.8% over our 12-week study period is also in line with estimates from highly affected areas in other European countries [2, 5–7]. The slight decrease in seroprevalence in the final weeks of our study may be due to several factors. One is sampling variation, with previously SARS-CoV-2-infected individuals likely having a higher motivation and fewer reservations to participate in the study during the lockdown, which took place at the beginning of our study. Also, the mail recruitment of participants without a known email address began in the second month and concerned mostly older individuals. However, we now know that clusters play an important role in the transmission dynamics of COVID-19 [25]. Thus, clustering of the cases in the sampled households might help to explain this variation. Similar observations have been made in a large serosurvey in the UK (REACT2), in which the seroprevalence declined from 6.0% to 4.4% between June and September 2020 [3].

Consistent with previous serosurveys [2], our study shows a significantly higher risk of infection in men (relative risk (RR) 1.22, 95% CrI 1.06–1.40) than in women. This should be considered when reviewing available evidence of increased complication and mortality rates in men [26]. Regarding age, we found higher relative risks of infection in adults of working age, teenagers and preadolescents than in children and older adults. While advanced age is now widely recognised as a risk factor for COVID-19-related complications and mortality [27], the relationship between age and susceptibility to infection probably depends on multiple factors. An immunological cause cannot be excluded at this stage. However, our study, like other serosurveys, was conducted in part during a lockdown. As such, it is likely that the measures in place affected adults, children and the elderly differently. The latter could have adopted and applied social isolation and preventive measures more strictly out of fear of suffering from severe complications, resulting in a decreased infection rate. Also, school closures may have contributed to the low seroprevalence observed in young children in our study, although recent household analyses based on our data suggest that children may be at reduced risk of infection even if exposed [28]. Adults of working age, on the other hand, could have had difficulty effectively socially distancing, as they were likely to be the ones to do grocery shopping and other tasks requiring them to leave the house. They might also have been essential workers, as evidenced by the fact that only a minority of participants (43.0%) reported working remotely since the beginning of the pandemic.

Odds of seropositivity were significantly reduced for retired women and unemployed men compared to employed women and men, respectively, adjusting for age. Seroprevalence was also lower, although with borderline significance, for all retired and unemployed individuals. These individuals could have had more flexibility in their daily routine allowing them to self-isolate better during the epidemic. While educational level was not significantly associated with the risk of infection, we did see trends of this association, except for doctoral-level education. It might be difficult to detect significant differences in the risk associated with educational level because it is linked to the profession of individuals in each educational level, which can be quite heterogenous. Finally, no significant association was found between median living area-based income and SARS-CoV-2 seropositivity.

Similar to previous reports, we found that current smokers were less likely to be seropositive [5]. Several hypotheses exist, including the effects of smoking on the expression of the angiotensin-converting enzyme 2 receptor in cells of the respiratory tract and the competition between nicotine and SARS-CoV-2 for the nicotinic acetylcholine receptor (nACfR), which acts as a co-receptor for viral cell entry [29]. Further work is needed to understand the behaviours that may contribute to this association, such as potential risk-averting behaviour in smokers out of a fear of respiratory complications of COVID-19.

Confirming previous reverse transcriptase polymerase chain reaction (rt-PCR)-based studies [30], in our sample, anosmia was frequently reported in seropositive (44%) but rarely in seronegative participants (3%), with individuals reporting this symptom being almost 25 times more likely to be seropositive than those who did not. However, as previously reported [31, 32], the correlation was weaker among individuals aged 65 years and older, possibly because the senses of taste and smell tend to decrease with age, thus making the symptom less discriminant [33, 34].

A recent meta-analysis based on rt-PCR diagnosis estimated the proportion of asymptomatic individuals infected with SARS-CoV-2 to be 20% (95% CI 17–25%), with a prediction interval of 3–67% [35]. However, due to the short window of the relatively high sensitivity of rt-PCR, it fails to capture all asymptomatic individuals. Also, individuals considered asymptomatic at the time of rt-PCR are potentially just presymptomatic [36]. Serological data mostly elude these difficulties if the delay between symptoms and testing is long enough. In our sample, 13% of IgG-positive individuals reported being asymptomatic. However, the number of participants with an asymptomatic SARS-CoV-2 infection in our sample is likely to be underestimated. Indeed, as the question referred to multiple past symptomatic episodes, it is possible that some of the symptoms reported by seropositive participants were non-SARS-CoV-2 related. Of note, 57% of seronegative participants reported one or more symptoms.

Our study found some age-related differences in terms of clinical presentation. In individuals younger than 18 years, only fever, cough and systemic symptoms were associated with seropositivity, whereas in adults, all symptom groups were significantly associated with seropositivity. Also, while being asymptomatic reduced the odds of seropositivity almost fivefold in the overall study population, odds were only twice reduced in children, with a higher percentage of asymptomatic positive children than positive adults (37% vs. 10%, Supplemental Table 11). Future serosurveys may be warranted in the context of new prevaling SARS-CoV-2 variants, in order to assess their impact on COVID-19 clinical presentation.

Our study comes with a number of limitations. The Bus Santé source population originally contained individuals between 20 and 74 years old, explaining the lower number of participants aged less than 18 and over 75 years in our final sample, despite the inclusion of primary participants’ household members. Efforts were made to increase the proportion of elderly individuals in our sample by offering home visits to those who were physically unable to travel to the testing centre or who preferred not to leave their homes because of their vulnerability to SARS-CoV-2. However, this population is still underrepresented in our sample. Also, the institutionalised older population was not included in the study. Furthermore, it is possible that individuals who believed they had been infected may have been more likely to participate, especially in early phases of the study, leading to a risk of selection bias. The seemingly low participation rate of 42% is a common issue in population-based surveys which do not use convenience samples (e.g. blood donors). Other SARS-CoV-2 serosurveys in high-income countries using a general population random sampling design have resulted in similar participation rates ranging from 19.5% to 53% [37–40]. Due to the urgent context in which the study took place during the first pandemic wave, our assessment of socioeconomic position was based on the most common indicators used, such as occupation and education [41, 42]. Further research should conduct more detailed analyses taking into account indicators better tailored to specific age groups (e.g. wealth, household conditions or parents’ education). Further, the association of income level with a seropositive status could not be assessed in the same manner as other socioeconomic factors, as we did not collect individual participants’ income, but estimated it based on their place of residency. One additional limitation was the large number of associations tested. Although this was not adjusted for, most significant associations held at *P* values less than 0.001. Memory bias likely influenced symptoms analysis, as participants were asked to report symptoms having occurred up to 6 months earlier. Also, some of the symptoms reported by IgG-positive participants may have been caused by other pathogens, as 1644 participants reported multiple episodes. Finally, the severity of the disease was not addressed in this study.

## Conclusions

Our study determined that the average seroprevalence of SARS-CoV-2 in the canton of Geneva over the course of the first wave was 7.8 %, leaving most of the population naive to the virus. Our risk factor analysis indicates that while some socioeconomic disparities in susceptibility to the infection do exist, they are likely to be more complex than what we previously thought. Older individuals seemed to be protected from infection during the first wave, perhaps due to targeted preventive measures. Some important observations could be made regarding differences in symptoms among age groups, such as the proportion of asymptomatic infections. However, future serosurveys should focus on larger samples of young children and the elderly to yield more detailed age-specific results regarding symptoms.

The results of our study can help inform global policy-makers as to how population immunity evolved after the first wave of COVID-19 in a highly affected area. Assuming similar preventive measures are kept in place, our results suggest that it will likely take time to reach herd immunity without risking the collapse of health systems. Moreover, this article adds to the still modest evidence base on risk factors for SARS-CoV-2 infection, and thus can help target public health interventions. Finally, the study of symptoms is an important step to refine diagnostic criteria and assist clinicians in their fight against the pandemic.

As sufficient vaccination of the population will likely take time, repeated seroprevalence surveys will be crucial to monitor the progression of the epidemic. Following up seropositive individuals will allow for determining the extent of the immune protection conferred by specific antibodies against re-infection by SARS-CoV-2, as well as provide insight into the prevalence of ‘long COVID’. Also, better characterisation of the sociodemographic risk factors for infection is needed to improve prevention strategies further. These measures will be of paramount importance to guide public health policy in order to manage future outbreaks of COVID-19 effectively.

## Supplemental Material

sj-docx-1-sjp-10.1177_14034948211048050 – Supplemental material for Seroprevalence of anti-SARS-CoV-2 IgG antibodies, risk factors for infection and associated symptoms in Geneva, Switzerland: a population-based study Supplemental material, sj-docx-1-sjp-10.1177_14034948211048050 for Seroprevalence of anti-SARS-CoV-2 IgG antibodies, risk factors for infection and associated symptoms in Geneva, Switzerland: a population-based study by Aude Richard, Ania Wisniak, Javier Perez-Saez, Henri Garrison-Desany, Dusan Petrovic, Giovanni Piumatti, Hélène Baysson, Attilio Picazio, Francesco Pennacchio, David De Ridder, François Chappuis, Nicolas Vuilleumier, Nicola Low, Samia Hurst, Isabella Eckerle, Antoine Flahault, Laurent Kaiser, Andrew S. Azman, Idris Guessous and Silvia Stringhini in Scandinavian Journal of Public Health
